# Poly(ADP-ribose) Polymerase 1, PARP1, modifies EZH2 and inhibits EZH2 histone methyltransferase activity after DNA damage

**DOI:** 10.18632/oncotarget.24291

**Published:** 2018-01-24

**Authors:** Lisa B. Caruso, Kayla A. Martin, Elisabetta Lauretti, Michael Hulse, Micheal Siciliano, Lena N. Lupey-Green, Aaron Abraham, Tomasz Skorski, Italo Tempera

**Affiliations:** ^1^ Fels Institute for Cancer Research & Molecular Biology, Lewis Katz School of Medicine at Temple University, Philadelphia, PA, USA; ^2^ Department of Pharmacology, Center for Translational Medicine, Lewis Katz School of Medicine at Temple University, Philadelphia, PA, USA; ^3^ Department of Microbiology and Immunology, Lewis Katz School of Medicine at Temple University, Philadelphia, PA, USA; ^4^ Present address: The Wistar Institute, Philadelphia, PA, USA; ^5^ Present address: Department of Microbiology and Immunology, Penn State College of Medicine, Hershey, PA, USA

**Keywords:** PARP1, EZH2, DNA damage, synthetic lethality, cancer therapy

## Abstract

The enzyme Poly(ADP-ribose) polymerase 1 (PARP1) plays a very important role in the DNA damage response, but its role in numerous aspects is not fully understood. We recently showed that in the absence of DNA damage, PARP1 regulates the expression of the chromatin-modifying enzyme EZH2. Work from other groups has shown that EZH2 participates in the DNA damage response. These combined data suggest that EZH2 could be a target of PARP1 in both untreated and genotoxic agent-treated conditions. In this work we tested the hypothesis that, in response to DNA damage, PARP1 regulates EZH2 activity. Here we report that PARP1 regulates EZH2 activity after DNA damage. In particular, we find that EZH2 is a direct target of PARP1 upon induction of alkylating and UV-induced DNA damage in cells and *in vitro*. PARylation of EZH2 inhibits EZH2 histone methyltransferase (H3K27me) enzymatic activity. We observed in cells that the induction of PARP1 activity by DNA alkylating agents decreases the association of EZH2 with chromatin, and PARylation of histone H3 reduces EZH2 affinity for its target histone H3. Our findings establish that PARP1 and PARylation are important regulators of EZH2 function and link EZH2-mediated heterochromatin formation, DNA damage and PARylation. These findings may also have clinical implications, as they suggest that inhibitors of EZH2 can improve anti-tumor effects of PARP1 inhibitors in BRCA1/2-deficient cancers.

## INTRODUCTION

The enzyme Poly(ADP-ribose) polymerase I (PARP1) is a well-known player in the DNA damage response [1]. PARP family members alter the function of target proteins by adding negatively charged polymers of ADP-ribose in an enzymatic reaction referred to as PARylation [2, 3]. When activated, PARP1 PARylates a number of target proteins involved in DNA damage repair, including PARP1 itself, transcription factors [4], chromatin modifiers [5, 6] and histones H1, H2 and H3 [7, 8]. The presence of long, negatively charged ADP-ribose polymers significantly alters target proteins. For example, the PARylation of histones decreases their affinity for DNA, due to electrostatic repulsion, and results in a more relaxed chromatin structure [9–12] and greater opportunities for gene expression [4, 13]. Although PARP1 activation is required for the DNA damage response, exactly why, and what activated PARP1 is necessary for, are not fully understood. The strict requirement for activated PARP1 in the DNA damage response suggests that additional PARP1 targets, especially targets intimately linked to the DNA damage response, remain to be identified.

Recent work from our laboratory showed that under normal physiological conditions (in the absence of DNA damage) PARP1 regulates the expression of the chromatin-modifying enzyme EZH2 [14]. EZH2 is a member of the polycomb repressive complex 2 (PRC2), a chromatin modifier that mediates the trimethylation of histone H3 at lysine 27 (termed H3K27me3) [15], which is associated with chromatin compaction and gene silencing. Specifically, we reported that inhibition of PARP catalytic activity with the PARP inhibitor olaparib resulted in global gene dysregulation, affecting approximately 11% of genes expressed in lymphoblastoid cell lines [14]. To identify the genes regulated by PARP, we conducted gene ontology analysis, which revealed that the genes dysregulated by PARP inhibition were primarily transcription factors and chromatin-remodeling enzymes, including EZH2. Inhibition of PARP activity as well as knockdown of PARP1 induced expression of EZH2. This upregulation of EZH2 had the expected functional consequence of increasing global H3K27me3. Together, these data identified for the first time a direct role of PARP1 in reducing the expression of EZH2, and thereby lowering EZH2 histone methyltransferase activity.

As noted above, this earlier work from our laboratory was done in conditions without DNA damage. Work from other groups has somewhat recently shown that EZH2 participates in the DNA damage response [16, 17]. In particular, Wang et al. reported that EZH2 can be phosphorylated in response to DNA damage and EZH2 activity regulates DNA damage-mediated apoptosis in T-cells, demonstrating a cross-talk between epigenetics and DNA repair [18]. Interestingly, Chou et al. showed that EZH2 and other members of the PRC2 complex are recruited to sites of DNA damage in a PARP-dependent manner [19]. Given these combined data, it seemed plausible that EZH2 could be a target of PARP1 not only under normal conditions, but also under conditions of DNA damage, the canonical condition when PARP1 is known to be activated and important for DNA repair. The concept that PARP1 might directly regulate the activity of EZH2 in response to UV-induced DNA damage had to our knowledge, not been considered.

The objective of this study was to test the overall hypothesis that, in response to DNA damage, PARP1 regulates EZH2 activity. We hypothesized that increased PARP activity that occurs during DNA damage causes PARP1 to PARylate EZH2, leading to decreased EZH2 activity and thereby decreased histone methyltransferase (H3K27me) activity. Since PARP1 is also known to PARylate histone H3, the substrate of EZH2, we hypothesized that PARylation of histone H3 may alter the affinity of EZH2 for its substrate. This study uses *in vitro* and cell-based biochemical methods to evaluate the interactions between PARP1, EZH2 and histone H3, the PARylation status of EZH2 and histone H3, EZH2 histone methyltransferase activity and changes in the affinity of EZH2 for its substrate H3.

We show that in response to DNA damage, PARP1 regulates EZH2 activity. These data are in accordance to recently published by Yamaguchi and colleagues [20]. Specifically, we find that EZH2 is a direct target of PARP1 upon induction of DNA damage in cells and *in vitro*. PARylation of EZH2 inhibits EZH2 histone methyltransferase (H3K27me) enzymatic activity. Induction of PARP1 activity by DNA alkylating agents decreases the association of EZH2 with chromatin, and PARylation of histone H3 reduces EZH2 affinity for H3. These findings establish that PARP1 and PARylation are important regulators of EZH2 function and link EZH2-mediated heterochromatin formation, DNA damage and PARylation. These findings may also have clinical implications because olaparib, a PARP inhibitor, has recently been approved for treatment of BRCA-mutated cancers [21]. PARP1 inhibition by olaparib induces synthetic lethality in tumor cells deficient in homologous recombination, an essential DNA repair pathway [22–25]. Since patients often develop resistance to olaparib [26–28], our results suggest that PARP1 inhibitors might be combined with EZH2 inhibitors to improve cancer therapy.

## RESULTS

### PARP1 interacts with EZH2 and PARylates EZH2 following alkylating DNA damage

We previously reported that under normal physiological conditions (i.e. no DNA damage) PARP1 inhibition induces the expression of EZH2 and increases H3K27me3 levels in cells [14]. It is well known that PARP1 activity is necessary for DNA repair and that upon induction of DNA damage PARP1 modifies several proteins. Since PARP1 PARylates proteins important in the DNA damage response, and because it has been reported that EZH2 plays a role in DNA repair, we hypothesized that in response to DNA damage, PARP1 modifies EZH2. Here we investigated the role of PARP1 and PARylation on EZH2 in response to DNA damage.

**Figure 1:**
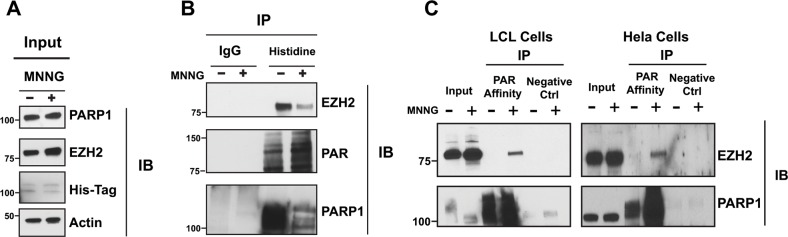
EZH2 interacts with PARP1 and is PARylated after DNA damage induction **(A)** Immunoprecipitation of EZH2 with PARP1 under physiological conditions and after induction of DNA damage. His-PARP1 was expressed in HeLa cells by transfection. Cells were treated with 100 uM of the alkylating agent N-methyl-N’-nitro-N-nitrosoguanidine (MNNG) for 10 minutes to induce DNA damage and activate PARP1. Input corresponds to 1/20^th^ of protein extracts from transfected cells used for the tag-construct pulldown. **(B)** Proteins interacting with EZH2 were analyzed by His pulldown or immunoprecipitated with non-immunogenic IgG (control) followed by western blot analysis with anti-EZH2 (top), anti-PAR (middle) and anti-PARP1 (bottom) antibodies. **(C)** Immunoprecipitation of EZH2 with PAR-affinity resin after induction of DNA damage. LCLs and HeLa cells were treated with or without 100 uM MNNG for 10 minutes. Cellular protein extracts were immunoprecipitated with a PAR affinity resin or PAR negative control resin and analyzed by western blot with anti-EZH2 and anti-PARP1 antibodies. Input corresponds to 1/10^th^ the amount of cell extracts used for immunoprecipitation.

We determined whether PARP1 associates with EZH2 under both physiological conditions and following DNA damage induction. We transfected HeLa cells with His-tagged PARP1 and induced DNA damage by treating cells with the alkylating agent N-methyl-N’-nitro-N-nitrosoguanidine (MNNG). We then identified proteins that interact with PARP1 by His-pull down. As a control we immunoprecipitated proteins with a non-immunogenic IgG. We assessed the interaction between PARP1 and EZH2 by western blot using antibodies specific for EZH2 and PARP1 (Figure 1B). We found that EZH2 co-precipitated with PARP1 but not with IgG. Overall, these results demonstrate that PARP1 and EZH2 form a complex in mammalian cells.

Recent studies from Izhar and colleagues demonstrate that several proteins, including transcription factors and chromatin-modifying enzymes, are recruited at sites of DNA damage in a PARP-dependent manner [29]. Upon DNA damage, PARP1 is activated and interacts with and modifies target proteins. Since we observed that PARP1 and EZH2 interact, we next investigated if EZH2 is PARylated following DNA damage induction. We induced DNA damage in lymphoblastoid cells (LCLs) and in HeLa cells by treating the cells with the alkylating agent MNNG. After 10 minutes, we isolated PARylated proteins by PAR-pull down using a PAR-affinity resin. After trapping the proteins on the PAR-resin, we assessed the PARylated proteins for EZH2 by western blot. We found that EZH2 was present in MNNG treated cells but not in the untreated cells or in the negative control resin (Figure 1C). We confirmed that the PAR-resin efficiently trapped PARylated proteins by assessing PARP1 trapping. PARP1 was isolated by the PAR-resin and treatment with MNNG increased the amount of purified PARP1, consistent with increasing PARP1 PARylation induced by DNA damage. These data show that EZH2 is PARylated upon DNA damage induction in LCLs and HeLa cells. Taken together, the data presented in Figure 1 show that EZH2 and PARP1 interact and that EZH2 is PARylated upon DNA damage induction.

### PARylation of EZH2 by PARP1 inhibits EZH2 histone methyltransferase activity

**Figure 2:**
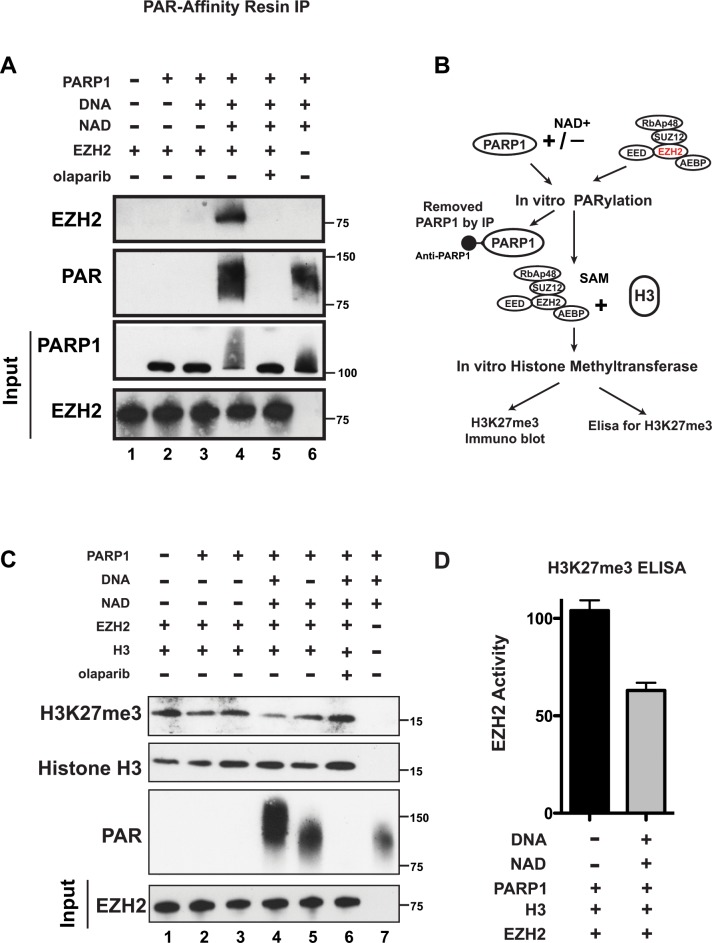
PARP1 PARylates EZH2 and inhibits EZH2 activity *in vitro* **(A)** PARylation of EZH2 by PARP1 *in vitro*. Human EZH2/PRC2 complex (EZH2, EED, SUZ12, RbAP48 and AEBP2) was incubated alone (lane 1) or with the agents indicated at the top (250 nM of olaparib (PARP inhibitor) was used and NAD+ is necessary for PARP1 activity). After 1 hour, PARylation was blocked by adding olaparib to all samples and PARylated proteins were pulled-down by PAR-affinity resin and analyzed by western blot with anti-EZH2 and anti-PAR antibodies. PARylation appears as a smear due to the different sizes of the various PAR polymers. Input corresponds to 1/10^th^ the amount of protein used for immunoprecipitation. Input was immunoblotted with anti-PARP1 and anti-EZH2 antibodies. **(B)** Schematic of the experimental strategy for C) and D). Briefly, the EZH2/PRC2 complex was incubated with PARP1 in the presence or absence of NAD+ as in A). After 1 hour, PARylation was stopped with olaparib and PARP1 was removed by immunoprecipitation with an anti-PARP1 antibody. The EZH2/PRC2 complex was incubated with EZH2 substrates histone H3 and S-adenosyl methionine (SAM) to allow histone methylation to occur. After 30 minutes, histone methytransferase activity was determined by assessing H3K27me3 levels. **(C)** *In vitro* histone methyltransferase assay. As indicated in B), purified histone H3 and SAM were incubated with the agents indicated at the top. After 30 minutes, proteins were analyzed by western blot using anti-Histone H3, anti-H3K27me3 and anti-PAR antibodies. Input corresponds to 1/20^th^ the amount of the protein used for immunoblotting. Input was probed with an anti-EZH2 antibody. **(D)** Levels of EZH2 activity with (black) and without (grey) PARP1 activity. Extracts from the EZH2/PRC2 complex incubated with histone H3 and SAM as in lanes 2 and 4 from C) were assessed for H3K27me3 levels by ELISA. N=3 ± SD.

We observed that EZH2 interacts with PARP1, suggesting that PARP1 may be responsible for EZH2 PARylation following DNA damage induction. Since DNA damage activates both PARP1 and PARP2, we next determined whether PARP1 PARylates EZH2 by assessing EZH2 PARylation by PARP1 *in vitro*. We incubated purified EZH2 and PARP1 in the presence or absence of the PARP substrate NAD+ to allow PARylation to occur (without NAD+ PARylation cannot occur because NAD+ supplies the ADP-ribose units that PARP attaches to proteins). After 1 hour, we immunoprecipitated the samples with PAR-affinity resin and probed the PARylated immunoprecipitates with an anti-EZH2 antibody. We detected PARylation of EZH2 only when NAD+ was present in the reaction (lane 4) (Figure 2A). We observed no PARylation of EZH2 when EZH2 was incubated with either PARP1 alone (lane 2) or with PARP1 and NAD+ in presence of the PARP inhibitor olaparib (lane 5) (Figure 2A). As a positive control for PARylation, we analyzed the PAR-resin pull-down by western blot with an anti-PAR antibody. We observed PARylation only when NAD+ was added to the reaction (lanes 4 and 5). We observed no PARylation when PARP1 and NAD+ were incubated in presence of olaparib (lane 5). PARylation of PARP1 was also detected, as shown by a smear of high molecular weight protein (lanes 4 and 5). These results reveal that EZH2 can be PARylated by PARP1.

PARylation can alter the functions of target proteins. Since EZH2 is necessary for the methylation of lysine 27 of histone H3, we investigated whether PARylation of EZH2 affects EZH2 histone methyltransferase activity. To determine this, we compared K27 tri-methyl levels of purified histone H3 incubated with PARylated EZH2 or unmodified EZH2 (as diagrammed in Figure 2B). Since EZH2 normally methylates lysine 27 of histone H3 as part of the PRC2 complex, we used a commercially available EZH2/PRC2 complex. We incubated the EZH2/PRC2 complex with PARP1 in the presence or absence of the PARP substrate NAD+, performed *in vitro* PARylation, and then added EZH2/PRC2 substrates S-adenosylmethionine (SAM) and purified histone H3 to allow methylation of lysine 27 of histone H3 to occur *in vitro*. Assessment of H3K27me3 levels by western blot (Figure 2C) showed that H3K27me3 decreased when EZH2 was incubated with PARP1 in the presence of NAD+ (allowing PARylation to occur) (lanes 4 and 5), compared to H3K27me3 levels when the EZH2/PRC2 complex was incubated with histone H3 alone (lane 1). We also observed no effect on H3K27me3 levels when EZH2 was incubated with PARP1 alone (lanes 2 and 3) or with NAD+ and the PARP inhibitor olaparib (lane 6). We quantified the reduction in EZH2 activity after PARylation using an H3K27me3 ELISA assay (Figure 2D) and found that PARylation of EZH2 reduces its activity by ≥40%. These results indicate that PARylation reduces histone methyltransferase activity of EZH2, resulting in decreased H3K27me3 and confirm that PARP activity, not just PARP1/EZH2 interaction, is required for reduction in EZH2 function. Taken together, these results indicate that PARylation of EZH2 inhibits its enzymatic activity, resulting in decreased levels of H3K27me3.

### PARylation stably inhibits EZH2 activity

**Figure 3:**
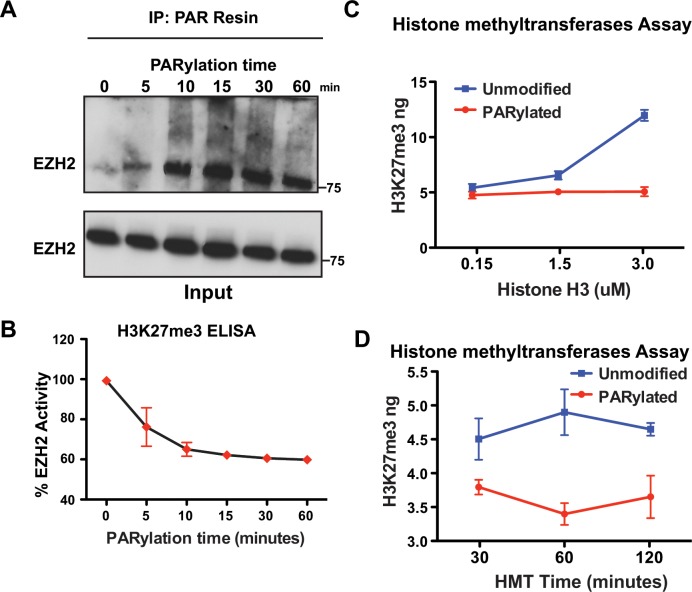
PARylation of EZH2 stably inhibits EZH2 enzymatic activity **(A)** Time course of *in vitro* PARylation assay. EZH2/PRC2 complex was incubated with PARP1, NAD+ and DNA fragments to allow *in vitro* PARylation. The reaction was blocked at different time points by adding the PARP inhibitor olaparib. After removing PARP1 from the reaction, PARylated proteins were pulled down with a PAR-affinity resin and analyzed by western blot with an anti-EZH2 antibody. Input corresponds to 1/10^th^ the amount of protein used for PAR pulldown. Input was probed with an anti-EZH2 antibody. **(B)** *In vitro* histone methylation assay. EZH2/PRC2 complex treated as in A) was incubated with histone H3 and SAM to allow methylation of lysine 27 of histone H3. After 30 minutes, histone H3 was extracted and H3K27me3 levels were measured by ELISA. EZH2 activity was calculated by setting H3K27me3 levels at time 0 as 100% EZH2 activity. N=3 mean ± SD. **(C)** *In vitro* histone methyltransferase activity assay. EZH2/PRC2 complex was incubated with PARP1 in the presence (PARylated) or absence (unmodified) of NAD+. After 1 hour, the reaction was blocked as in A) and EZH2/PRC2 complex was incubated with SAM and different concentrations of histone H3 to allow histone H3-K27 methylation to occur. After 30 minutes, the reaction was blocked and the amount of methylated histone H3-K27 generated by EZH2 activity was measured using an H3K27me3 ELISA kit. N=3, mean ± SD. **(D)** Time course of *in vitro* histone methyltransferase (HMT) activity. EZH2/PRC2 complex was treated as in C) and incubated with SAM and histone H3 to allow methylation of H3-K27. The reaction was blocked at different time points and the amount of methylated histone H3-K27 generated by EZH2 activity was measured by an H3K27me3 ELISA. N=3, mean ± SD.

The results presented in Figure 1 showed that EZH2 is PARylated after induction of DNA damage for 10 minutes in cells. To determine if EZH2 activity decreases immediately upon its PARylation, we assessed EZH2 PARylation *in vitro* over time. We incubated EZH2 and PARP1 together with NAD+ and blocked the reaction at different time points. We then purified PARylated proteins by PAR-resin pull-down and performed western blot analysis using an anti-EZH2 antibody (Figure 3A). We observed significant PARylation of EZH2 after 5 minutes of *in vitro* PARylation. We also found that EZH2 PARylation increased over time and reached saturation after 15 minutes. These results show that PARylation of EZH2 is a quick reaction that reaches saturation shortly after PARP activation. These observations are consistent with our results in cells showing PARylation of EZH2 immediately after DNA damage induction.

Next, we looked for a correlation between PARylation of EZH2 and its effect on EZH2 histone methyltransferase activity over time. EZH2/PRC2 histone methyltransferase activity was evaluated as in Figure 3B. We found that *in vitro* PARylation inhibited EZH2 activity over time, with a 20% reduction in activity after 5 minutes and a maximum reduction of 40% after 30 minutes, which remained constant after 60 minutes of PARylation. Inhibition of EZH2 activity correlated with the saturation of EZH2 PARylation occurring at the same time points. Taken together, these data indicate that inhibition of EZH2 activity is proportional to the extent of EZH2 PARylation, and that a significant reduction in EZH2 activity is achieved immediately after PARP1 activation.

In our *in vitro* experiments we assessed EZH2 activity by keeping both the concentration of histone H3 and the reaction time constant. However, *in vivo* neither histone H3 concentration nor histone methylation time are limiting conditions; thus, either may offset the inhibitory effect of PARylation on EZH2. To determine whether EZH2 activity could be rescued *in vitro* by either more substrate (H3) or more time, we assessed EZH2 activity in experimental settings that recapitulate the cellular environment. First, we incubated the EZH2/PRC2 complex with PARP1 as described in Figure 2A to allow for EZH2 PARylation. After removing PARP1, the EZH2/PRC2 complex was then incubated with different amounts of histone H3 for 30 minutes and H3K27me3 levels were measured by ELISA. We observed no change in H3K27me3 levels when more histone H3 was added to the EZH2/PRC2 complex when both PARP1 and NAD+ were present (Figure 3C, red line). On the contrary, H3K27me3 levels increased when more histone H3 was added to the EZH2/PCR2 complex incubated only with PARP1 (Figure 3C, blue line). These results show that EZH2 inhibition through PARP1 is unaffected by increased concentrations of histone H3.

Next, we investigated if longer incubation time with histone H3 could offset EZH2 inhibition by PARP1. We incubated the EZH2/PRC2 complex as described above with histone H3 to allow histone methylation to occur and measured H3K27me3 at different time points by ELISA (Figure 3D red line). We found that H3K27me3 did not increase when PARylated EZH2 was incubated with histone H3 for longer time, whereas H3K27me3 levels did increase over time when histone H3 was incubated with unmodified EZH2 (Figure 3D blue line). These data reveal that longer incubation with histone H3 does not offset inhibition of EZH2 by PARylation. Overall, the results presented in Figure 3 show that EZH2 activity is inhibited upon activation of PARP1 and increased amount of substrate or longer reaction time do not offset the inhibitory effect of EZH2 PARylation, suggesting that as long as EZH2 is PARylated its enzymatic activity is reduced.

### PARG removes PAR polymers from EZH2 restoring EZH2 enzymatic activity

**Figure 4:**
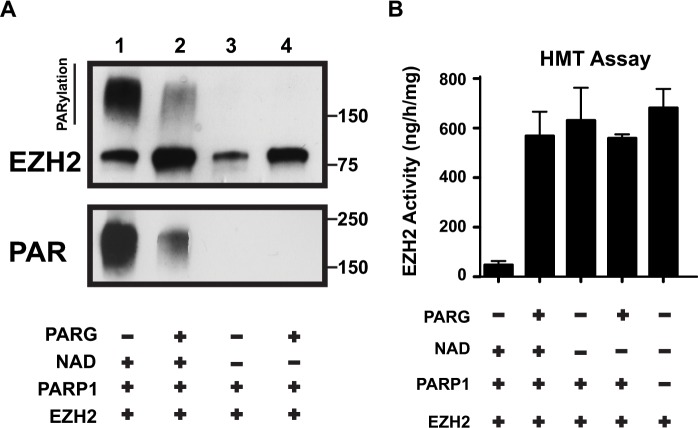
PARG reverses EZH2 PARylation and restores EZH2 enzymatic activity **(A)** *In vitro* PARG assay. EZH2/PRC2 complex and PARP1 were incubated with or without PARG as indicated (Note: NAD+ is required for PARylation). After 1 hour, the reaction was blocked by addition of the PARP inhibitor olaparib and the EZH2/PRC2 complex was incubated with (lanes 2 and 4) or without (lanes 1 and 3) PARG to allow degradation of PAR polymers. After 1 hour, the reaction was stopped by adding Laemmli buffer and the proteins were analyzed by western blot using anti-EZH2 and anti-PAR antibodies. The upper band in the top panel represents PARylated EZH2. PARG activity was confirmed by reduction of PAR smear. **(B)** *In vitro* histone methyltransferase (HMT) activity assay. EZH2/PRC2 complex treated as in A) was subsequently assayed for histone methyltransferase activity using an HMT assay kit. The activity of EZH2 under the indicated conditions was calculated based on the amount of H3-K27 converted in the assay. As a control, the activity of EZH2/PRC2 complex alone was also determined. N=3, mean ± SD.

*In vivo*, PARylation is a transient and reversible modification due to the action of poly(ADP-ribose) glycohydrolase, PARG [30]. PARG degrades PAR polymers through both endo- and exoglycosidase activities, releasing both PAR polymers and ADP-ribose monomers from modified proteins [31]. To further confirm that PARylation is responsible for reducing EZH2 activity, and to show that in this experimental system the effect of PARylation is reversible, we tested whether the catalytic activity of EZH2 can be rescued by PARG. We incubated EZH2 with PARP1 as described above and added PARG to the reaction after 1 hour to allow for degradation of PAR polymers. We assessed the effect of PARG on EZH2 by western blot using anti-EZH2 and anti-PAR antibodies. As expected, EZH2 PARylation significantly decreased after PARG addition (Figure 4A top panel) as indicated by the reduction in the smear of high molecular weight protein. Additionally, the degradation of PAR polymers into ADP-ribose monomers was confirmed (Figure 4A, lower panel). Thus, PARG de-PARylates EZH2. Next, we confirmed that removal of the PAR polymers from EZH2 by PARG was sufficient to restore EZH2 histone methylatransferase activity (Figure 4B). We found that incubating EZH2 with PARG restored the enzymatic activity of EZH2 even when PARP1 and NAD+ were present (lane 2 compared to lane 1). The results also confirmed the requirement for NAD+ as incubation of EZH2 with PARP1 alone did not reduce EZH2 activity (lane 3). Together these results demonstrate that PARylation inhibits EZH2 in a reversible manner and PARG activity can restore EZH2 function.

### PARylation of histone H3 inhibits EZH2 enzymatic activity

**Figure 5:**
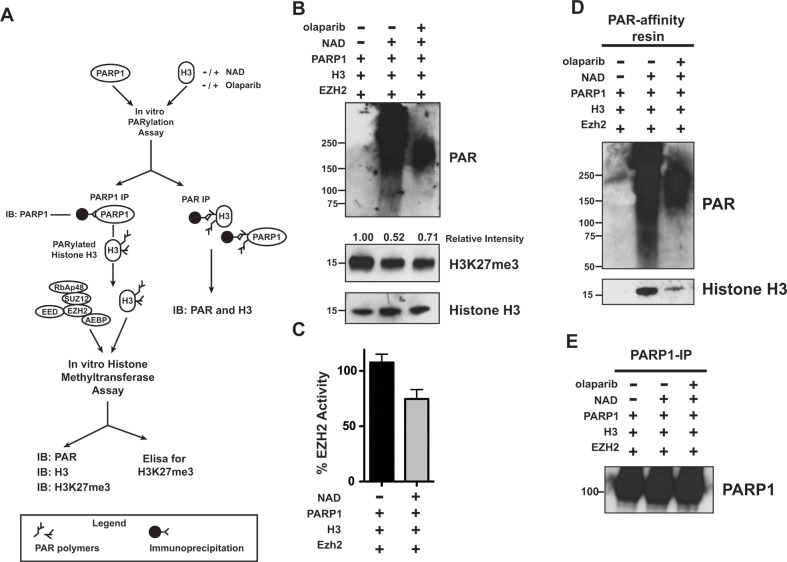
PARylation of Histone H3 decreases EZH2-mediated histone methylation **(A)** Schematic of experimental approach that couples histone PARylation and methylation *in vitro*. First, histone H3 was incubated with PARP1 in the presence or absence of NAD+ and olaparib to allow for PARylation. After 60 minutes, the reaction was blocked by addition of olaparib, PARP1 was removed by immunoprecipitation and the remaining histone H3 was either assessed for PARylation by PAR-resin pulldown or incubated with EZH2/PRC2 and SAM to allow H3-K27 methylation *in vitro*. After 30 minutes, the histone methyltransferase reaction was blocked and H3K27me3 levels were determined by different approaches. **(B)** Histone H3 PARylation decreases subsequent H3-K27 methylation. Histone H3 proteins treated as in A) were analyzed by western blot using an anti-H3K27me3 antibody and an anti-histone H3 antibody as a control. The signal intensity of H3K27me3 relative to H3 was measured using ImageJ software and normalized to the signal from unmodified histone H3 (H3 incubated with PARP1 in the absence of NAD+, lane 1). PARP1 activity was confirmed by western blot using an anti-PAR antibody. The western blot is representative of three independent experiments. **(C)** PARylation reduces histone methylation *in vitro*. Histone H3 samples treated as in A) were used to determine EZH2 activity toward unmodified and PARylated histone H3 by measuring H3K27me3 levels using an ELISA kit. H3K27me3 levels from unmodified histone H3 were set as 100% EZH2 activity. N=3, mean ± SD. **(D)** *In vitro* PARylation of histone H3. Histone H3 proteins treated as in A) were immunoprecipitated using the PAR-affinity resin and PARylation of H3 was confirmed by western blot using an anti-H3 antibody. PAR-resin specificity and PARylation levels were determined by western blot analysis of purified proteins with an anti-PAR antibody. The smear observed in lane 2 indicated PARylation. H. **(E)** PARP1 Immunoprecipitation. PARP1 removal after PARylation of histone H3 treated as above was confirmed by western blot analysis of proteins immunoprecipitated with an anti-PARP1 antibody.

We observed that following induction of DNA damage EZH2 is PARylated in cells (Figure 1). We confirmed these observations *in vitro* and further demonstrated that PARylation of EZH2 inhibits EZH2 activity, suggesting that upon DNA damage induction PARP1 could modify and inhibit EZH2 activity (Figure 2). However, following DNA damage induction *in vivo*, PARP1 induces the PARylation of several targets, including histone H3. Since histone H3 is also the substrate of EZH2, we tested if PARylation of histone H3 affects EZH2 interaction with H3 *in vitro*. We first incubated histone H3 with PARP1 in the presence or absence of NAD+ and with or without the PARP inhibitor olaparib. Next, we removed PARP1 from the reaction by immunoprecipitation using an anti-PARP1 antibody (as outlined in Figure 5A). We then incubated “PARP1-free” histone H3 with the EZH2/PRC2 complex to allow histone H3 methylation and evaluated EZH2 activity by assessing H3K27me3 levels by western blot (Figure 5B). We observed that H3K27me3 levels decreased when histone H3 was incubated with PARP1 and NAD+ (lane 2) compared to when histone H3 was incubated with PARP1 without NAD+ (lane 1). We also observed that inhibition of PARP1 activity partially rescued the inhibitory effect on EZH2, increasing H3K27me3 levels (Figure 5B, lane 3). We confirmed these observations and further quantified the inhibitory effects of histone H3 on EZH2 activity by measuring H3K27me3 levels under the same experimental conditions by ELISA (Figure 5C). We determined that PARylation of histone H3 decreased H3K27 methylation by ~30%, consistent with western blot analysis, although the magnitude of inhibition was slightly different probably due to differences in method sensitivity. To confirm that the observed reduction in EZH2 activity was due to histone H3 PARylation, we purified PARylated proteins by PAR-affinity resin pull-down and analyzed the precipitated proteins by western blot using anti-PAR and anti-histone H3 antibodies (Figure 5D). We found that histone H3 was PARylated only when it was incubated with PARP1 and NAD+ (lane 2) and that olaparib reduced histone H3 PARylation (lane 3). Finally, we confirmed that the histone methyltransferase assay was carried out in the absence of PARP1, which otherwise could also modify EZH2, by analyzing immunoprecipitated PARP1 by western blot using an anti-PARP1 antibody. We found that PARP1 was trapped on the beads and removed from the reaction (Figure 5E). Overall, these data reveal that PARylation of histone H3 inhibits EZH2-mediated histone methylation.

### PARylation affects EZH2 affinity for histone H3

**Figure 6:**
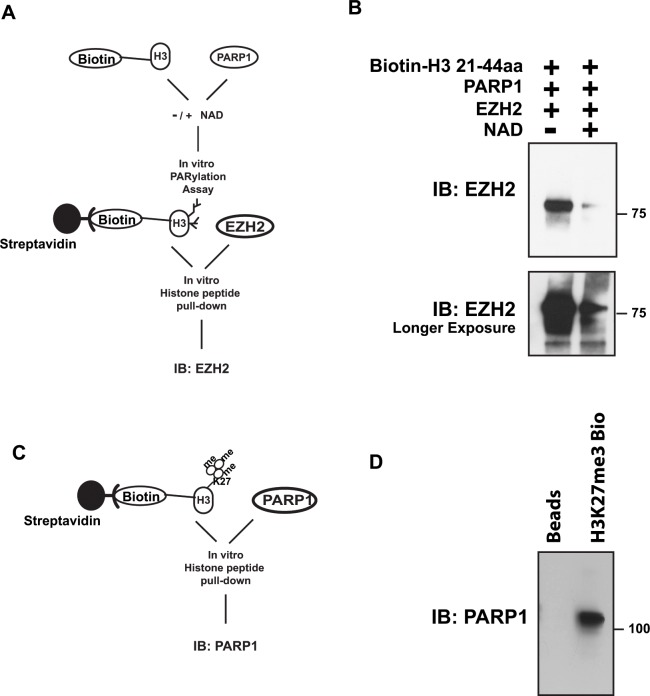
PARylation of histone H3 decreases EZH2 affinity for H3, while methylation of histone H3 has no effect on the ability of PARP1 to interact with histone H3 **(A)** Schematic of histone peptide pull-down after PARylation. **(B)** Histone peptide pull-down for EZH2. Synthesized histone H3 peptide, corresponding to residues 21-44 of human histone H3, was conjugated with biotin and incubated with PARP1 in the presence or absence of NAD+ to allow for PARylation. After 1 hour, the reaction was blocked with 250 nM olaparib and the H3 peptide was immunopurified with streptavidin-magnetic beads and subsequently incubated with EZH2/PRC2 complex. After 4 hours, the peptide-coated, streptavidin-conjugated beads were washed to remove unbound proteins and bound proteins were analyzed by western blot using an anti-EZH2 antibody. PARylation of the peptide was confirmed by western blot using an anti-PAR antibody. Top panel shows short film exposure; lower panel longer film exposure. **(C)** Schematic of histone peptide pull-down after *in vitro* histone methyltransferase assay. **(D)** Histone peptide pull-down assay for PARP1. Synthesized histone H3 peptide containing tri-methylated lysine 27 was conjugated with streptavidin magnetic beads followed by incubation with purified PARP1. After 4 hours, the peptide-coated, streptavidin-conjugated beads were washed to remove unbound proteins and bound proteins were analyzed by western blot using an anti-PARP1 antibody.

PARylation can alter the chemical and physical properties of its modified proteins, including affinity for other factors or substrates. Binding of EZH2 to histone H3 is necessary for the methylation of lysine 27 through EZH2 enzymatic activity. Since PARylation of histone H3 inhibits methylation of H3 by EZH2 (Figure 5), we asked whether PARylation of H3 affects the affinity of EZH2 for histone H3. We assessed if EZH2 binds to PARylated histone H3 by histone pull-down using a biotin-labeled histone peptide corresponding to residues 21-44 within human histone H3 (Figure 6A). Analysis of histone-associated proteins by western blot with an anti-EZH2 antibody (Figure 6B) revealed that EZH2 interaction with the histone peptide decreased when PARP1 and NAD+ were present. To determine if methylation of histone H3 affects the ability of PARP1 to interact with the histone, we performed a reciprocal experiment in which we assessed PARP1 interaction with the histone H3 peptide that contained tri-methylated lysine 27 as diagrammed in Figure 6C. Analysis of the histone-associated proteins (Figure 6D) by western blot with an anti-PARP1 antibody showed that PARP1 interacted with the histone peptide regardless of histone methylation status (Figure 6D). Taken together, these results suggest that PARylation of histone H3 decreases the affinity of EZH2 for histone H3 (its substrate), whereas methylation of histone H3 does not affect the ability of PARP1 to interact with histone H3.

### EZH2 association with chromatin decreases following PARP1 activation in cells

**Figure 7:**
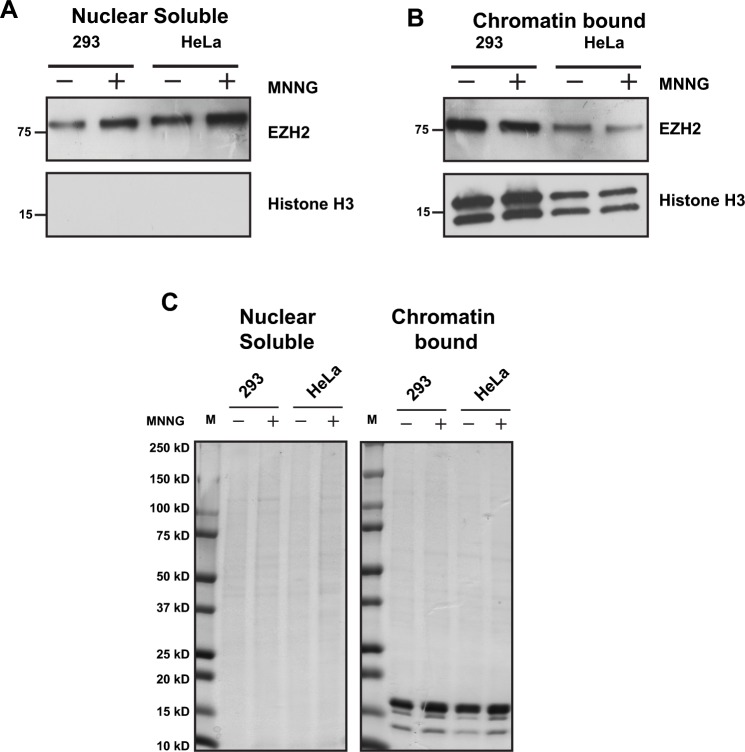
EZH2 interaction with chromatin decreases after DNA damage **(A and B)** EZH2 association with chromatin after DNA damage. HEK293 cells and HeLa cells were treated with or without 100 uM MNNG to induce DNA damage. After 10 minutes, proteins were extracted and fractionated to obtain nuclear soluble and chromatin-bound protein extracts. The fractionated proteins were analyzed for EZH2 expression by western blot using an anti-EZH2 antibody. The effectiveness of separation of nuclear soluble and chromatin-bound proteins was determined by western blot with an anti-histone H3 antibody. All western blots are representative of at least three independent experiments. **(C)** Global levels of nuclear soluble proteins and chromatin-bound proteins are unaffected by PARP1 activation. Nuclear soluble and chromatin-bound protein extracts from HEK293 cells and HeLa cells treated as in B) were separated by gel electrophoresis on a 4-20% polyacrylamide gel and stained with coomassie brilliant blue to confirm both correct fraction separation and equal protein quantity. The image is representative of at least three independent experiments.

The results in Figure 1 indicate that EZH2 is PARylated upon MNNG treatment, and the results in Figure 6 show that PARylation reduces EZH2 affinity for histone H3 *in vitro*. Therefore, we tested whether this was also the case in our cell-based systems. To determine whether induction of DNA damage reduced the association of EZH2 with chromatin in cells, we induced DNA damage in 293HEK and HeLa cells by MNNG treatment and assessed EZH2 levels in the nuclear soluble fraction and chromatin-bound fraction by western blot. In both cell lines we found that DNA damage increased the levels of EZH2 in the nuclear soluble fraction (Figure 7A) but decreased the amount of EZH2 associated with chromatin (Figure 7B). We observed no changes in total protein levels of both fractions after DNA damage induction (Figure 7C). These data reveal that EZH2 association with chromatin is reduced after DNA damage, which is consistent with our *in vitro* data (Figure 6) showing that PARylation reduces the affinity of EZH2 for histone H3. Overall, these data indicate that PARP1 activity influences EZH2 interaction with chromatin.

### EZH2 PARylation decreases global H3K27me3 levels after DNA damage

**Figure 8:**
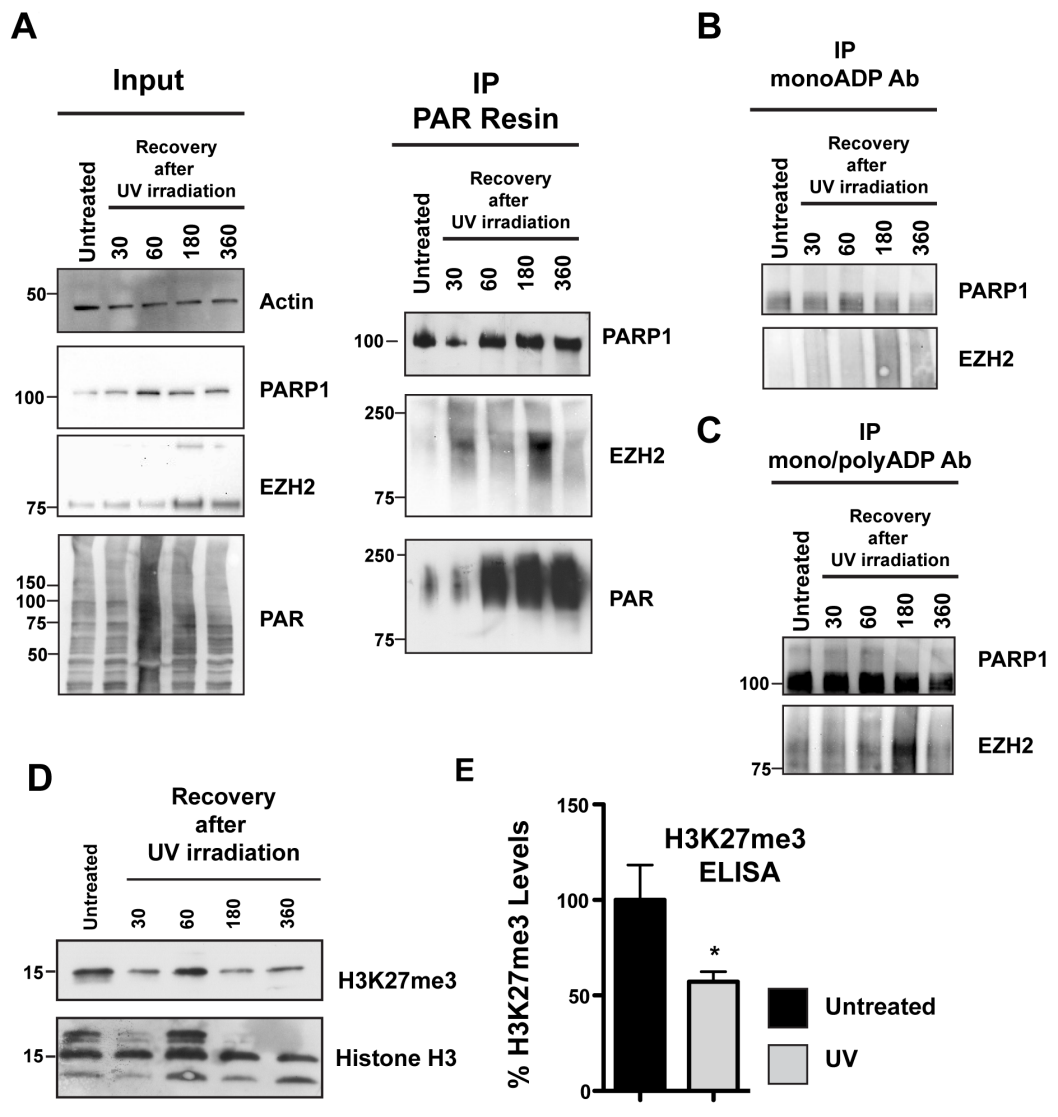
EZH2 PARylation decreases global H3K27me3 levels after DNA damage **(A)** PARylation of EZH2 after DNA damage by UV radiation. HeLa cells were irradiated with UVA and UVB for 2 minutes and then recovered in media. PARylated proteins were pulled down at the indicated recovery times using PAR-resin and analyzed by western blot using anti-PARP1, anti-EZH2 and anti-PAR antibodies. The shift of EZH2 to a higher molecular weight isoform indicates its PARylation. EZH2 PARylation increases and reaches a maximum at 3h after the initial treatment with UV. Input corresponds to 1/20th the amount of protein extract. **(B and C)** HeLa cells were treated as above and proteins were pulled down using either an anti mono-ADP-ribose antibody or an anti mono/poly-ADP-ribose antibody. Immunoprecipitated proteins were then analyzed by western blot with anti-EZH2 and anti-PARP1 antibodies. The western blot is representative of at least three independent experiments. Untreated cells served as control. **(D)** Histones were extracted from HeLa cells treated as described above. H3K27me3 levels were analyzed by western blot with an anti-H3K27me3 antibody or anti-histone H3 as control. **(E)** Quantification of H3K27me3 levels after UV irradiation-induced DNA damage. HeLa cells were exposed to UV as described in A) and recovered in media. After 3 hours, histones were purified and assessed for H3K27me3 levels by ELISA. The level of K27 methylation under the indicated conditions were calculated based on the amount of H3-K27 converted in the assay, divided by the amount of total histones loaded. Data were normalized to the untreated and expressed as % of H3K27me3. Data were N=3, mean ± SD. Statistically significant differences between experimental conditions and control samples were determined by Student's *t* test.

Our cellular data in Figure 1 indicate that EZH2 is PARylated upon induction of DNA damage by alkylation. Our *in vitro* data in Figure 2 show that PARylation of EZH2 represses EZH2 catalytic activity, and data reported in Figure 6 show that PARylation decreases EZH2 affinity for histone H3. Combining these pieces of data, we next tested if EZH2 function is altered by DNA damage. We used UV radiation as a source of DNA damage since it induces different types of DNA lesions and was also used in previous studies of EZH2 function [32]. We induced DNA damage in HeLa cells using UVA and UVB radiation and proteins were extracted at different recovery times. We isolated PARylated proteins by PAR-pull down using PAR-affinity resin as described above. After trapping proteins on the PAR-resin we assessed PARylated proteins for the presence of EZH2 by western blot. We found that EZH2 was present in UV treated cells but not in untreated cells (Figure 8B). We also observed that EZH2 PARylation increased over time and reached the highest PARylation level 3 hours (180 minutes) post UV exposure. Consistent with this observation, 3 hours after UV radiation we observed an EZH2 isoform with increased molecular weight in the input samples (Figure 8A) and is characteristic of PARylated proteins (Figure 8A). These data are consistent with our *in vitro* results showing that PARylation of EZH2 occurs following DNA damage.

Rouleau and colleagues reported that EZH2 can also interact with PARP3, another member of the PARP family that is involved in the DNA damage response [33]. PARP3 is closely related to PARP1 and PARP2 but is a mono-ADP-ribosylase [34]. We next assessed if EZH2 is mono-ADP ribosylated following DNA damage induction. We irradiated the cells with UV as described above and then isolated mono- or poly-ADP-ribosylated proteins by immunoprecipitation using antibodies specific for mono- (Figure 8B) or mono/poly-ADP ribose (Figure 8C). We assessed mono- and poly-ADP-ribosylated proteins for EZH2 by western blot. We observed no EZH2 mono-ADP ribosylation after induction of DNA damage (Figure 8B). We confirmed that the mono-ADP-ribose antibody efficiently trapped modified proteins by assessing PARP1 trapping. We found that PARP1 was isolated by the mono-ADP-ribose antibody and that treatment with UV slightly increased the amount of PARP1, consistent with changes in PARP1 mono-ADP-ribosylation after DNA damage induction. We found that EZH2 was among the proteins pulled down by the poly-ADP-ribose antibody in UV treated cells. As observed in the experiments using the PAR resin, we detected that EZH2 PARylation was highest 3 hours (180 minutes) post-UV exposure. As a control we confirmed that PARP1 was immunoprecipitated by the antibody against poly-ADP-ribose (Figure 8C). Taken together these data confirmed that following UV irradiation EZH2 is mostly PARylated.

Since EZH2 is PARylated after induction of DNA damage in cells and our data in Figure 3 indicate that PARylation inhibits EZH2 activity, we tested whether global levels of H3K27me3 change after UV irradiation. We treated the cells with UV as described above and extracted total histones. We assessed global H3K27me3 levels by western blot and found that H3K27me3 levels fluctuated in the first hour after UV radiation but steady decreased after 180 minutes of UV radiation (Figure 8D), in correspondence with increasing PARylation of EZH2. We confirmed that no significant changes in H3 levels occurred after UV radiation by western blot (Figure 8D). We also quantified the reduction in H3K27me3 levels after UV radiation by ELISA (Figure 8E). More specifically, we measured H3K27me3 levels after 3 hours of recovery post-irradiation by ELISA, which is when we observe the maximum level of PARylation of EZH2 by western blot. We found that H3K27me3 levels decreased after DNA damage induction, with a 50% reduction 3 hours post-treatment compared to the control (Figure 8E). Together these results demonstrate that PARylation inhibits EZH2 and decreases H3K27me3 levels after DNA damage induction.

### An EZH2 inhibitor increases the effect of a PARP1 inhibitor in BRCA-deficient cell lines and in AML patient cells

PARP1 inhibitors are used to induce synthetic lethality in cells and tumors deficient for DNA repair (i.e. BRCA1-deficient cells). Our data *in vitro* and in cell systems indicate that following DNA damage PARP1 modifies EZH2, which results in lower H3K27me3 levels. Chromatin organization regulated by histone modifications (e.g., tri-methylation of H3K27) plays an essential role in the accumulation and repair of DNA double-strand breaks (DSBs) [35]. In the context of synthetic lethality, global relaxation of chromatin could render cancer cells, but not normal cells, more susceptible to DSB formation and drug-induced genotoxic cell death [36]. We postulate that de-condensed chromatin may enhance the accumulation of DNA damage induced by genotoxic agents and DNA repair inhibitors, such as PARP1 inhibitors, thus promoting synthetic lethality.

**Figure 9:**
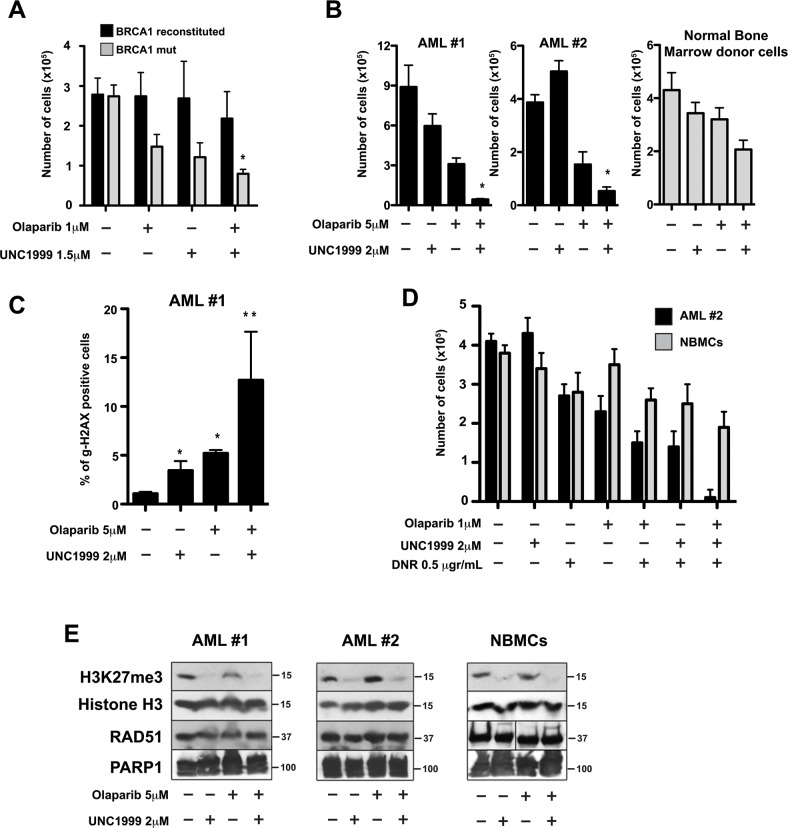
EZH2 inhibitor UNC1999 enhanced PARP1 inhibitor olaparib-mediated synthetic lethality in BRCA-deficient cell lines and acute myeloid leukemia (AML) primary cells **(A)** *BRCA1*-mutated and *BRCA1*-reconstituted MDA-MB-436 human breast carcinoma cells were treated with or without the PARP1 inhibitor olaparib in the presence or absence of the EZH2 inhibitor UNC1999 at the indicated concentrations. After 4 days, cell count/viability was determined by Trypan blue exclusion using a Bio Rad TC20 Automated Cell Counter. Results show mean ± SD of living cells. N=3. ^*^p<0.05 compared to individual treatment using Student *t* test. **(B)** Lin-CD34^+^ AML primary cells from two patients and from healthy bone marrow donor (NBMCs) were treated with 2μM UNC1999, 5 μM olaparib, or were left untreated. After 4 days, live cell number was determined by Trypan blue exclusion using a Bio Rad TC20 Automated Cell Counter. Results show mean × SD number of live cells. N=3. ^*^p<0.05 compared to individual treatment using Student *t* test. **(C)** Lin-CD34^+^ AML primary cells (patient #1) were treated with olaparib and UNC1999 as indicated. After 48 hours, DNA damage was evaluated by measuring the percentage of γ-H2AX-positive Lin-CD34^+^ AML#1 cells by flow cytometry. Data are mean × SD. N=3. ^*^p<0.001 in comparison to Control; ^**^p<0.05 in comparison to UNC1999 or olaparib treatment. **(D)** Lin-CD34^+^ AML #2 cells and NBMCs were isolated as in B) and treated with UNC1999, 0.5 μg/μl DNR, 1 μM olaparib, the indicated drug combinations, or were left untreated. After 4 days living cells were determined by Trypan blue exclusion assay. Data are mean × SD. N=3; ^*^p<0.01 compared to individual/dual treatment using Student *t* test. **(E)** Proteins were extracted from Lin-CD34^+^ AML cells and NBMCs after 2 days of treatment with the indicated drug combination and analyzed by western blot with antibodies specific for H3K27me3, histone H3, RAD51 and PARP1 proteins. Blots are representative of three independent experiments.

Since PARP1 inhibition induces accumulation of DNA damage in BRCA1-deficient cells, we tested if inhibition of EZH2 enhances the effect of the PARP inhibitor olaparib in BRCA1-mutated and BRCA1-reconstituted MDA-MB-436 human breast carcinoma cells [37, 38]. We treated the cells with or without olaparib in the presence of UNC1999, a potent inhibitor of EZH2 (as well as EZH1 with an IC50 of 2 nM and 45 nM in cell-free assays, respectively) [39]. As previously reported, we found that PARP inhibition reduced the growth of BRCA1-mutated cells (Figure 9A). We also observed that the EZH2 inhibitor reduced the growth of BRCA1-mutated cells compared to BRCA1-reconstituted cells (Figure 9A). When we treated the cells with both inhibitors we found that EZH2 inhibition increased the effects of olaparib against the BRCA1-mutated cells, whereas BRCA1-reconstitued cells were not affected. These data suggest PARP1 inhibition coupled with EZH2 inhibition could enhance the effects of synthetic lethality for cancer treatment.

To test if EZH2 inhibition enhanced the effect of olaparib in primary BRCA-deficient tumor cells, we treated Lin^−^CD34^+^ cells from patients with BRCA-deficient AML and from healthy donors with olaparib with or without UNC1999. We found that the EZH2 inhibitor UNC1999 greatly increased the effect of PARP inhibitor olaparib against BRCA-deficient Lin-CD34^+^ AML cells, whereas normal bone marrow counterparts were only modestly sensitive (Figure 9B). Next, we tested if the combination of UNC1999 with olaparib increased DNA damage induced by olaparib. We assessed DNA damage by γH2AX, a well-known marker for DNA DSBs, by immunofluorescence in Lin-CD34^+^ AML cells treated as above. We found that UNC1999 enhanced the accumulation of DSBs induced by olaparib in BRCA-deficient Lin^−^CD34^+^ AML cells (Figure 9C).

AML patients are usually treated with daunorubicin, a DNA intercalating agent that induces DNA lesions. Since we observed that combination of UNC1999 and olaparib increased olaparib-induced DSBs in AML cells, we tested whether UNC1999 increases the effect of daunorubicin (DNR) + olaparib. We found that UNC1999 enhanced the effect of DNR + olaparib by 15-fold in BRCA-deficient AML cells, but normal cells were only modestly affected (Figure 9D). We confirmed that UNC1999 treatment lowered H3K27me3 levels without changing the expression of PARP1 or RAD51 (a key member of BRCA-mediated homologous recombination) proteins (Figure 9E).

Taken together, our results indicate that the combination of the EZH2 inhibitor UNC1999 and the PARP1 inhibitor olaparib enhances DSB formation and cell death induced by olaparib in cells deficient in DNA repair pathways.

## DISCUSSION

In this study we investigated the role of PARP1 in regulating EZH2 functions in the context of DNA damage. We previously reported that under normal physiological conditions PARP1 activity regulates EZH2-mediated chromatin repression [14]. Recent work reported that EZH2 is also involved in DNA repair and is recruited at the site of damage in a PARP-dependent fashion [19]. Whether PARP1 activity alters EZH2 function not only under normal physiological conditions, but also in the context of DNA damage, had not been fully considered.

In this work we discovered that, upon induction of DNA damage, EZH2 is PARylated by PARP1 and PARylation inhibits EZH2 histone methyltransferase activity, resulting in reduced H3K27me3. We also found that PARylation of histone H3 inhibits EZH2 activity by reducing the affinity of EZH2 for its substrate, histone H3. Together, our results suggest that PARylation of EZH2 and/or histone H3 can affect EZH2 enzymatic activity following DNA damage induction. These findings demonstrate that PARP1 is an important regulator of EZH2 functions and indicate that a crosstalk exits between PARP activity and heterochromatin formation in the context of DNA damage. While we were preparing this manuscript, Yamaguchi and colleagues reported similar observations about the interaction and the effect of PARP1 activity on EZH2 [20], confirming and supporting the results outlined here. Our work also confirms that EZH2 plays a role in DNA repair and indicates that EZH2/PARP1 interaction plays an important and underappreciated role in repairing DNA. Our findings may have significant translational implications since aberrant EZH2 activity contributes to cancer and PARP1 inhibitors are in clinical trials, and we show that combinatorial treatment with an EZH2 and PARP1 inhibitor enhances PARP-mediated genotoxicity in cells from cancer patients.

Our findings that PARP1 interacts with, PARylates, and inhibits EZH2 activity upon DNA damage supports the hypothesis that one of the major functions of PARP1 activation during DNA damage is to open chromatin structure to allow DNA repair [9–12]. In support of this hypothesis, Strickfaden et al. recently showed that after inducing DNA damage by laser microirradiation, chromatin is decondensed and histones are displaced in a PARP-dependent manner [40]. Our data and recently published work further suggest that in the context of DNA damage, inhibition of EZH2 activity by PARP1 may be necessary to prevent the PRC2 from condensing chromatin structure, which would impair the repair of DNA.

Several studies showed that EZH2 and other members of the PRC2 and PRC1 complexes are recruited to sites of DNA damage and that EZH2 is necessary for the repair of DNA breaks [41, 42]. However, how EZH2 (and PRC2) contribute to repairing DNA damage is still not fully understood. For example, Choud et al. and O'Hagan et al. observed that H3K27me3 levels increase at DNA damage sites [17, 19], whereas Campbell et al. and Sustáčková et al. reported no changes in H3K27me3 levels at sites of DNA damage [32, 43]. These differences could be due to the fact that the groups were looking at different types of DNA damage generated by different insults (ionizing radiation compared to alkylating agents) and suggest that EZH2 activity could be differentially regulated by different pathways of DNA repair. Nevertheless, our observations that PARylation inhibits EZH2 activity during DNA damage could explain the lack of H3K27me3 accumulation at sites of damage, despite the observed recruitment of EZH2 to damaged DNA sites. The recruitment of EZH2/PRC2 at sites of DNA damage without an increase in H3K27me3 suggests that EZH2/PRC2 are able to regulate DNA repair in a histone methyltransferase-independent mechanism. In this scenario, PARP1 activity is critical to ensure that EZH2/PRC2 function to repair DNA (by an unknown mechanism) and to shield the DNA damage site from EZH2-mediated chromatin condensation.

Our data are consistent with a role for EZH2 in DNA repair that involves changes in H3K27me3, as we observed that immediately after UV radiation H3K27me3 levels decrease then return to normal, even though PARP1 activity increased. H3K27me3 levels then drop significantly after a few hours. The kinetics of this response to DNA damage induction (no steady changes at early times; significant changes at later times) could reflect differences between the role of EZH2 in DNA repair, which occurs immediately after damage, and the role of EZH2 in repressing gene expression, which may occur a few hours after the DNA insult. Our findings support a role of PARP1 activation in regulating both functions of EZH2. The immediate early inhibition of EZH2 by PARP1 prevents EZH2-mediated de-novo histone methylation and chromatin condensation; the later inhibition of EZH2 by PARP1 may prevent EZH2 from repressing genes involved in DNA repair. The latter hypothesis is consistent with: a) the reported observation that EZH2 represses the expression of RAD51, which is involved in DNA repair through the homologous recombination pathway [44, 45]; and b) with our previous work showing that PARP activity blocks EZH2-mediated gene repression [14].

EZH2 can methylate other substrates beside histones [46, 47]. Therefore, it may be possible that in the context of DNA repair, PARylation of EZH2 reduces the methylation of histones, but has no effect on the non-histone targets of EZH2. It would be interesting to determine whether other targets of EZH2 play a role in DNA repair. Nevertheless, our findings show that EZH2 is a target of PARP1 activity and indicate that PARP1 activation could be an important regulator of EZH2 functions during DNA repair.

Our work also provides insight into the role of PARylation in regulating EZH2. One potential effect of PARylation is to decrease the affinity of EZH2 for its substrate, histone H3. We observed that EZH2 PARylation correlates with a global decrease in the association of EZH2 with chromatin. Since previous work showed that EZH2 is recruited at the site of DNA damage, our observation that DNA damage reduces EZH2 association with chromatin suggests that only low amounts of EZH2 are required at the site of damage, as observed for other proteins involved in DNA repair and consistent with the observation from Campbell et al, described above [32]. The release of EZH2 from chromatin could also be due to the PARylation of histone H3, as our data show that unmodified EZH2 binding is reduced when histone H3 is PARylated. It is interesting to note that, somewhat conversely, methylation of histone H3 does not change PARP1 affinity for the histone. Decreased association between EZH2 and PARylated histone H3 and the inhibition of histone methylation of PARylated EZH2 suggest that PARP1 activity regulates the affinity of EZH2 for the histone H3. It has very recently been reported that PARylation of EZH2 also alters the affinity of EZH2 for the other members of the PRC2 complex, affecting PRC2 stability. Our results complement and extend previous work indicating PARylation of EZH2 inhibits EZH2 activity by disassembling the PRC2 complex [20]. Whether such destabilization occurs besides the DNA damage response remains an open question that we are planning to address. Nevertheless, our results are consistent with the possibility that the role of EZH2 in DNA repair is independent of its well-characterized histone-modifying activity and may involve an unknown function of EZH2. It is possible that EZH2-histone methyltransferase activity could impair DNA mending by condensing chromatin at the site of DNA damage, a process prevented by PARP1 and PARylation of EZH2.

Our reported observation that PARP1 and EZH2 interact after DNA damage bear important translational consequences as PARP1 inhibitors are currently used to treat cancer. EZH2 is overexpressed in several cancers, including lymphomas and breast cancer [48–50], and overexpression of EZH2 correlates with resistance to cisplatin in ovarian cancer [51]. Our data showing that EZH2 inhibition greatly enhanced the genotoxic effect of the FDA approved PARP inhibitor olaparib on hemapoietic cells from AML patients support the proposed hypothesis of a role of EZH2 in DNA repair. Based on our data and the literature, we envision that EZH2 inhibitors may improve the efficacy of PARP1 inhibitors for new therapeutic approaches, as well as for current cancer treatment, leading to a more precise use of these classes of compounds.

In summary, our results, together with previous work from our group and others, reveal a new function of PARP1 activity in the DNA damage response by showing that PARP1 and PARylation are important mechanisms of EZH2 regulation. Our work adds an important branch to the existing model of how PARP1 activity regulates chromatin in the context of DNA damage, and how two epigenetic factors, EZH2 and PARP1, communicate to regulate gene expression through chromatin modification. Gaining a better insight into the PARP1/EZH2 interaction will reveal new important molecular steps that are necessary for cells to repair DNA.

## MATERIALS AND METHODS

### Plasmids and antibodies

The plasmid encoding His-fused full length human PARP1 was a gift from John Pascal and described elsewhere [52, 53]. The plasmid encoding the HA-fused full length human EZH2 plasmid (pCMVHA hEZH2) was a gift from Kristian Helin (Addgene plasmid # 24230) [54]. The plasmid encoding GST-fused full length human EZH2 (Pgex-EZH2) was a gift from Mien-Chie Hung (Addgene plasmid # 28060) [55]. All plasmids were verified by sequencing. Rabbit polyclonal anti-His (WB 1/200, Ip: 2.5 μgr/sample), rabbit anti-Mouse IgG HRP conjugated (WB: 1/5000; IP 2.5 μgr/sample), and mouse anti-Rabbit IgG HRP conjugated (WB: 1/5000, IP: 2.5 μgr/sample) were from Santa Cruz Biotechnology. Monoclonal mouse anti-HA (WB 1/1000) was from Origene. Monoclonal mouse anti-HA (IP 2.5 μgr/sample) and rabbit polyclonal anti-Histone H3 (WB 1/2000) were from Abcam. Rabbit polyclonal anti-PARP1 (WB 1/10000) and rabbit polyclonal anti-H3K27me3 (WB 1:2000) were from Active Motif. Rabbit polyclonal anti-PAR antibody (WB 1/1000) was from Trevigen. Mouse monoclonal anti-EZH2 antibody (WB 1/2000; IP 2.5 μgr/sample) was from BD Bioscience. Rabbit polyclonal anti-Actin antibody (WB 1/100) was from Sigma Aldrich. Anti-mono-ADP-ribose binding reagent and Anti-mono- and poly-ADP-ribose binding reagent (IP 5 μgr/sample) were from Millipore.

### Cell culture and plasmid transfection

Cell lines were maintained in a humidified atmosphere containing 5% CO_2_ at 37°C. LCLs were cultured in suspension in RPMI 1640 supplemented with fetal bovine serum at a concentration of 15%. HEK293 and HeLa cells were cultured in Dulbecco's modified Eagle's medium (DMEM) supplemented with 10% FBS. *BRCA1*-mutated and *BRCA1*-reconstituted MDA-MB-436 human breast carcinoma cells were obtained from Neil Johnson (Fox Chase Cancer Center) [37]. All cell media was supplemented with 1% penicillin/streptomycin. LCLs, HeLa and HEK293 cells were treated with 100 μM N-Methyl-N’-nitro-N-nitrosoguanidine (MNNG; Pfaltz & Bauer) for 10 minutes immediately prior to harvesting. Plasmids for His-PARP1 and HA-EZH2 were received as *E. coli* glycerol stocks. Upon receipt, Luria broth was inoculated with the stock, a culture was grown overnight at 37°C shaking and plasmids were extracted using the Pureyield Plasmid Midiprep System (Promega). All transfections were performed using Lipofectamine 2000 (Invitrogen) according to the manufacturer's instructions for either 6-well or 10-cm format.

### Western blot analysis, immunoprecipitation, his pulldown and PAR pulldown

For western blotting of endogenous proteins and tagged protein constructs cells were lysed in RIPA buffer (50 μM Tris-HCl, pH 7.4, 150 μM NaCl, 0.25% deoxycholic acid, 1% NP-40, 1 μM EDTA; Millipore) supplemented with 1X protease inhibitor cocktail (Thermo Scientific). Protein extracts were obtained by centrifugation at 3,000×*g* for 10 minutes at 4°C. For nuclear fractionation, nuclear soluble and chromatin-bound protein fractions were extracted from 2×10^6^ cells using the Subcellular Protein Fractionation Kit for Cultured Cells (Invitrogen) according to manufacturer's instructions. The bi, cinchoninic (BCA) protein assay (Pierce) was used to determine protein concentration. Lysates were boiled with 1X Laemmli sample buffer (Bio-Rad) supplemented with 1.25% β-mercaptoethanol (Sigma-Aldrich). Protein extracts were resolved by gel electrophoresis on a 4–20% polyacrylamide gradient Mini-Protean TGX precast gel (Bio-Rad) and transferred to an Immobilon-P membrane (Millipore) for western blotting analysis. Membranes were blocked in 5% milk in PBS-T for 1 hour at room temperature, and then incubated with indicated primary antibodies.

For analysis of histone proteins from cells histone proteins were acid extracted from 2×10^6^ cells following the histone extraction protocol from Abcam and quantified by BCA protein assay. 1 μgr of histones was analyzed by SDS-PAGE followed by western blot or by an H3K27me3 ELISA kit (Active Motif) according to manufacturer's protocol. For analysis of purified recombinant histone H3 protein (Active Motif C110A) from *in vitro* reactions, histone peptide was acid extracted from the reaction as described above.

For co-immunoprecipitation 1×10^7^ cells were used per IP. Cells were resuspended in 1 mL NET buffer (50 μM Tris–HCl, 150 μM NaCl, 5 μM EDTA, 0.5% NP-40 pH 7.4), sonicated (30% output) six times using a sonic dismembrator (Fisher Scientific) to shear cells and DNA, and protein extracts were obtained by centrifugation at 10,000×*g* for 5 minutes at 4°C. The supernatant was then incubated with 2.5 μg of indicated antibodies overnight at 4°C followed by incubation with 100 mL of 50% Protein A/G Sepharose beads (Thermofisher). After 2 hours incubation at 4°C with rotation, the beads were washed three times with NET Buffer - low salt – (50 μM Tris–HCl, 150 μM NaCl, 5 μM EDTA, 0.5% NP-40 pH 7.4) and three times with NET Buffer - high salt – (50 μM Tris–HCl, 300 μM NaCl, 5 μM EDTA, 0.5% NP-40 pH 7.4) then resuspended in Laemmli buffer and analyzed by SDS-PAGE and western blotting.

For His Pulldown, 1×10^7^ cells were resuspended in NET buffer as described above for immunoprecipitation, and protein extracts were incubated with 50 mL His-magnetic beads for 2 hours. The beads were washed with NET buffer as described above and resuspended in Laemmli buffer and analyzed by SDS-PAGE and immunoblotting.

For PAR pulldown, 2×10^7^ cells were resuspended in 1 mL of PAR Lysis buffer [50 μM Tris, pH 8, 200 μM NaCl, 1 μM EDTA, 1% Triton X-100, 10% glycerol, 1 μM DTT, 0.5% deoxycholate, 1X protease inhibitors (Thermo Scientific), 1 μM ADP-HPD (Adenosine 5’-diphosphate (hydroxymethyl) pyrrolidinediol) (EnzoLifesciences)] and incubated for 2 hours at 4°C with rotation. Proteins were then extracted by centrifugation at 3000x*g* for 5 minutes at 4°C. 500 mL of the protein extracts were then incubated with 20 mL (20 μg) of either Poly-ADP-ribose (PAR) Affinity resin (Tulip BioLabs, 2302) or Poly-ADP-ribose (PAR) Negative Control Resin (Tulip BioLabs, 2303). The PAR Affinity resin is a purified GST-Af1521 macrodomain fusion protein construct. The Af1521 macrodomain has been shown to bind with high affinity polymeric ADP-ribose modified proteins. The PAR Negative Control resin is identical to the PAR positive except it contains a mutated Af1521 macrodomain that is unable to bind PAR. After overnight incubation at 4°C with rotation, beads were washed three times with PAR Lysis buffer and resuspended in 80 mL Laemmli buffer and incubated at 65°C for 15 minutes to dissociate the macrodomain fusion protein from affinity-precipitated proteins. 30 mL of purified PARylated proteins were then analyzed by SDS-PAGE and immunoblotting. For PAR pulldown of proteins from *in vitro* reactions, samples were resuspended in 500 mL of PAR buffer and incubated with either positive or negative PAR-affinity resin as described above.

### *In vitro* PARylation assay

Recombinant proteins (500 ng) were incubated in 20 mL PAR reaction buffer containing 0.5 U of human PARP1 enzyme (Tulip, #2090), 50 μM Tris (pH 8.0), 10 μM MgCl_2_, 5 μM KCl, with or without 1 μM NAD^+^, and with or without 1 μg/mL activated DNA (Sigma D4522). When indicated, 250 nM of the PARP inhibitor olaparib (Selleckshem) was added to the reaction. Reactions were incubated for 60 minutes at RT. For time course experiments the reaction was incubated for 5, 10, 15 or 30 minutes. The PARylation reactions were immediately inactivated by the addition of 2x SDS-PAGE loading buffer followed by SDS-PAGE and Western blot analysis. For PAR-pulldown and other reactions following *in vitro* PARylation, PARP1 was inactivated by adding 250 nM olaparib (except for samples in which the inhibitor was previously added) to the reaction and the samples were kept on ice for 30 minutes.

### *In vitro* PARG assay

Samples subjected to *in vitro* PARylation as described above were incubated in 20 mL PARG reaction buffer containing 20 μM KPO, pH 7.5, 5 μM KCl, and with or without 10 ng human PARG (Trevigen). The reaction was incubated for 1 hour at 37°C. For immediate analysis of PAR hydrolysis the reaction was inactivated by the addition of 2x SDS-PAGE loading buffer followed by SDS-PAGE and Western blot analysis. For the histone methyltransferase assay following the PARG assay, PARG was inactivated by adding 250 nM ADP-HPD (Adenosine 5’-diphosphate (hydroxymethyl) pyrrolidinediol) (Enzo Life Sciences) to the reaction and the sample was kept on ice. After 30 minutes, samples were incubated at RT for 10 minutes and the enzymatic activity of EZH2/PRC2 complex was measured by the EpiQuick Histone Methyltransferase Activity/Inhibition assay (EpiGentek) according to manufacturer's protocol.

### *In vitro* histone methyltransferase assay

After PARylation, the human EZH2/EED/SUZ12/RbAp48/AEBP2 Complex (BPS Bioscience) was incubated in 30 mL HMT buffer containing 50 μM Tris, pH 8, 10 μM MgCl_2_, 10 μM DTT, and 3 μg purified Histone H3 (Active Motif, C110A) with or without 40 μM [S-(5’-Adenosyl)-L-methionine] (SAM) (BPS Bioscience). For western blot analysis of H3K27me3 levels, the reaction was inactivated after 30 minutes by the addition of 2x SDS-PAGE loading buffer followed by SDS-PAGE and immunoblotting. For ELISA analysis of H3K27me3 levels, the reaction was inactivated after 30 minutes by acid extraction of histone H3 following the histone extraction protocol from Abcam. Histone H3 was then quantified by BCA and H3K27me3 levels were measured by Histone H3 trimethyl Lys27 ELISA kit (Active Motif) according to manufacturer's protocol. For time course experiments, the histone methyltransferase reaction was carried out for either 30, 60 or 120 minutes.

### Quantification of PAR levels in cellular extracts

Cellular PAR levels were quantified using the PARP *in vivo* Pharmacodynamic Assay 2^nd^ Generation (PDA II) kit (Trevigen) according to the manufacturer's protocol. Briefly, 3×10^6^ cells were lysed in the provided buffer, and protein concentration was determined with the BCA protein assay (Pierce). Cell extracts were added to the provided PAR capture plate and incubated overnight at 4°C. Wells were washed four times with phosphate-buffered saline containing 0.05% Tween-20 (PBS-T) and incubated with the polyclonal PAR detection antibody at room temperature for 2 hrs. After washing four times with PBS-T, extracts were incubated with goat-anti-rabbit IgG-HRP antibody at room temperature for 1 hr. Wells were washed four times with PBS-T before adding the PARP PeroxyGlow reagent and luminescence was measured using a POLARstar Optima microplate reader (BMG Labtech).

### Histone peptide pull-down

6 μg of biotinylated Histone H3 peptide (residues 21-44) (EpiGentek) was subjected to either *in vitro* PARylation or an *in vitro* histone methyltransferase assay as described above. After the appropriate time (see above for details) 400 mL of PBS containing either a PARP1 or EZH2 inhibitor (250 nM olaparib or 50 nM GSK343, respectively) were added to the reaction. The reaction was then incubated with 20 mL of streptavidin-coupled magnetic beads (Themofisher) at 4°C with rotation. After 1 hour, the beads were collected using a magnet and washed three times with wash buffer (PBS, 150 μM NaCl (final salt concentration at 300 μM), 0.1% Triton X-100, 2 μM DTT, 1X protease inhibitor cocktail). The peptide-bound beads were then resuspended in 400 mL of incubation buffer (PBS, 0.1 % Triton X-100, 2 μM DTT, and 1X protease inhibitor cocktail) containing 10 μg of either PARP1 or the EZH2/PRC2 complex. The peptide-bound beads and proteins were incubated for 4 hours at 4°C with rotation and washed three times with wash buffer as described above. The washed beads were resuspended in 40 mL 1X Laemmli buffer and boiled at 95°C for 5 minutes and analyzed by SDS-PAGE followed by immunoblotting. For PARP1 pull down, 6 μg of synthesized biotin-conjugated trimethyl histone H3-K27 peptide derived from residues 1-100 of human histone H3 (EpiGentek) was incubated for 30 minutes with 20 mL of streptavidin-coupled magnetic beads, washed several times with wash buffer then resuspended in 400 mL of incubation buffer containing 10 μg of purified PARP1. After 4 hours, the peptide-bound beads were treated as described above and analyzed by SDS-PAGE followed by immunoblotting.

### *In vitro* UV irradiation

HeLa cells were irradiated with the Junggust Box, a hand-made enclosed module that houses two F20 sunlamps outputting 60% UVB and 38% UVA wavebands. A total UV dose of 175 Jm-2 was given at a dose rate of 2.0 Wm-2. The cells were irradiated in 10-cm culture dishes at 80% confluence. Following 2 minutes of irradiation, the cells were incubated for the desired recovery time in DMEM supplemented with 10% FBS and 1% penicillin/streptomycin.

### EZH2/PARP1 inhibition in BRCA-deficient cells

Primary cells from peripheral blood and bone marrow samples from patients with BRCA-deficient AML were previously described [37, 38]. Normal hematopoietic cells were purchased from Cambrex Bio Science (Walkersville, MD, USA). Lin^−^CD34^+^ cells were obtained from mononuclear fractions by magnetic sorting using the EasySep negative selection human progenitor cell enrichment cocktail followed by the human CD34 positive selection cocktail (StemCell Technologies) as previously described [38, 56]. Cells were incubated with olaparib, UNC1999 and/or daunorubicin (DNR) (all from Selleckchem) in IMDM supplemented with 10% FBS and growth factors (100 ng/μl SCF, 20 ng/μl IL-3, 100 ng/μl Flt-3 ligand, 20 ng/μl G-CSF, 20 ng/μl IL-6). Cell viability was counted by trypan blue exclusion test as described before [38, 56]. For detection of protein expression, nuclear cell lysates were obtained as described before [57] and analyzed by Western blot using anti-H3K27me3 (EMD Millipore, 07-449) and anti-H3 (Abcam, ab61251) antibodies. Cell viability and γH2AX were measured by flow cytometry using Fixable Viability Dye eFluor® 660 (eBioscience) and Alexa Fluor® 488 Mouse anti-H2AX (pS139) (BD Pharmingen) as described before [56]. For DSB detection a rabbit polyclonal antibody against histone H2A.X phosphorylated at Ser193 (#613403, Biolegend) was used and flow cytometry analysis was performed using the LSR Fortessa (Becton Dickinson) as previously described [38].

### Study approval

Studies involving were approved by the appropriate Institutional Review Boards and met all requirements of the Declaration of Helsinki.

### Statistical analysis

All experiments presented were conducted at least in triplicate (three biological replicates and three technical replicates for each experiment) to ensure reproducibility of results. The Prism statistical software package (GraphPad) was used to identify statistically significant differences between experimental conditions and control samples using Student's *t* test as indicated in the figure legends.

## Abbreviations

PARP: (Poly(ADP-ribose) polymerase 1)
PAR: Poly(ADP-ribose)
PARG: Poly(ADP-ribose) glycohydrolase
EZH2: Enhancer Of Zeste 2 Polycomb Repressive Complex 2
PRC1: Polycomb Repressive Complex 1
PRC2: Polycomb Repressive Complex 2IR
LCLs: lymphoblastoid cell lines
